# Supplementary figures and images for: Susceptibility of rabbits to SARS-CoV-2
Source: Emerg Microbes Infect. 2021 Jan 17;10(1):1–7. doi: 10.1080/22221751.2020.1868951 (PMC7832544; doi:10.1080/22221751.2020.1868951)

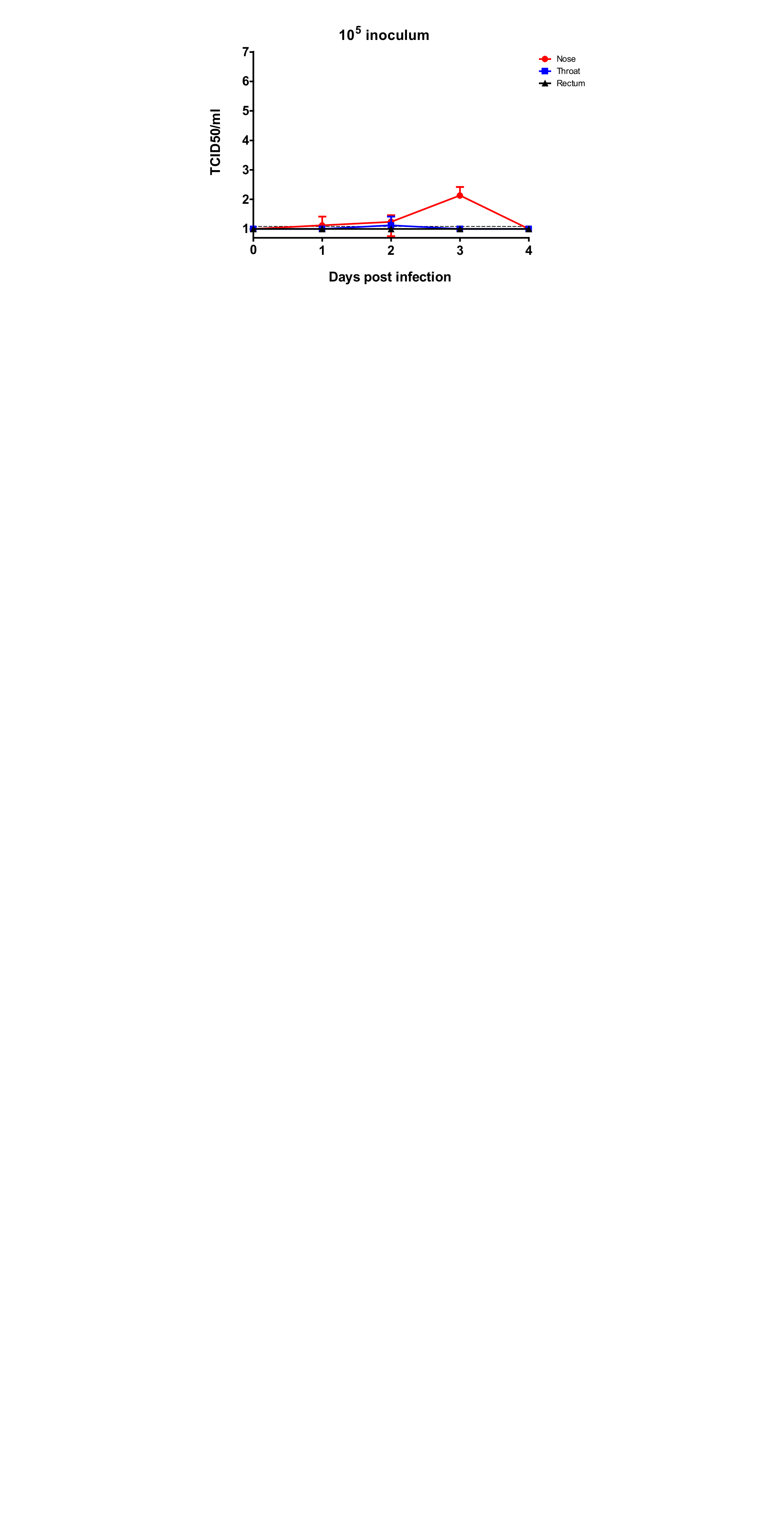

Supplement: 201210_Revised_EMI_Figures_no_fig_legends-4.tiff [file TEMI_A_1868951_SM7376.tiff]
